# Comment on ‘Weight loss in heart failure is associated with increased mortality only in non‐obese patients without diabetes’ by Niedziela *et al*.

**DOI:** 10.1002/jcsm.12649

**Published:** 2020-11-26

**Authors:** Nadja Scherbakov, Wolfram Doehner

**Affiliations:** ^1^ Berlin‐Brandenburg Center for Regenerative Therapies (BCRT) Charité—Universitätsmedizin Berlin Berlin Germany; ^2^ German Center for Cardiovascular Research (DZHK), partner site Berlin Berlin Germany; ^3^ Department of Cardiology Charité—Universitätsmedizin Berlin Berlin Germany; ^4^ Center for Stroke Research Berlin (CSB) Charité—Universitätsmedizin Berlin Berlin Germany

Niedziela *et al*. reported in their recent paper ‘Weight loss in heart failure is associated with increased mortality only in non‐obese patients without diabetes’ in the journal that the presence of diabetes abolishes the impact of weight loss (WL) on mortality.^1^ Over the past decades, WL is a highly discussed issue within the scientific community. As a serious complication, WL accompanies a wide range of clinical disorders including cancer, heart failure, stroke, and chronic kidney disease and contributes to poor prognosis.^2^, ^3^, ^4^, ^5^ The most severe form of the WL, cachexia, has been recognized as a life‐threatening disorder that has been recorded in the ICD‐10 diagnosis code as R64. Diabetes mellitus (DM) is a frequent co‐morbidity in patients with heart failure and is associated with increased hospitalization rate and mortality.^6^ Combination of both diabetes and cachexia is often observed in heart failure. However, until now, there have been no clinical studies investigating WL and mortality in heart failure patients with vs. without DM.

In the present study, the authors retrospectively investigated an impact of WL on 1 year survival of diabetic and non‐diabetic patients with heart failure with reduced ejection fraction (HFrEF). In the entire patient cohort, a lower mortality rate was observed only in patients without significant WL (<7.5% of body weight, stable weight, or weight gain) and without DM compared to other three patients groups (−WL + DM, WL − DM, and WL + DM). This result provides additional evidence that co‐morbidities such as DM and WL contribute independently to poor clinical outcome in patients with heart failure.

In further analyses, they observed no differences in mortality between obese patients with or without WL and with or without DM. No difference in 1 year survival was shown in non‐obese patients with or without WL when DM was present DM. The WL was established as an independent predictive factor of 1 year mortality in non‐diabetic patients without obesity prior the onset of HFrEF. Thus, the present study provides first evidence that the presence of DM or the presence of obesity eliminates an impact of WL on clinical outcome in a subgroup of patients with HFrEF.

We have previously shown in patients with DM or with prediabetes and a wider range of cardiovascular co‐morbidity^7^, ^8^ that WL in these patients was associated with increased mortality. Further studies are warranted to explore why the co‐morbidity of heart failure and DM seems to attenuate this effect. The author certifies that he complies with the ethical guidelines for publishing in the Journal of Cachexia, Sarcopenia and Muscle: update 2019.^9^
